# Silica fertilization improved wheat performance and increased phosphorus concentrations during drought at the field scale

**DOI:** 10.1038/s41598-021-00464-7

**Published:** 2021-10-21

**Authors:** Jörg Schaller, Eric Scherwietes, Lukas Gerber, Shrijana Vaidya, Danuta Kaczorek, Johanna Pausch, Dietmar Barkusky, Michael Sommer, Mathias Hoffmann

**Affiliations:** 1 “Silicon Biogeochemistry” Working Group, Leibniz Centre for Agricultural Landscape Research (ZALF), 15374 Müncheberg, Germany; 2 University of Bayreuth, 95440 Bayreuth, Germany; 3 “Isotope Biogeochemistry and Gas Fluxes” Working Group, Leibniz Centre for Agricultural Landscape Research (ZALF), 15374 Müncheberg, Germany; 4 “Landscape Pedology” Working Group, Leibniz Centre for Agricultural Landscape Research (ZALF), 15374 Müncheberg, Germany; 5 “Experimental Infrastructure Platform”, Leibniz Centre for Agricultural Landscape Research (ZALF), 15374 Müncheberg, Germany; 6 Institute of Geography and Environmental Science, University of Potsdam, 14476 Potsdam, Germany

**Keywords:** Biogeochemistry, Ecology, Environmental sciences

## Abstract

Drought and the availability of mineable phosphorus minerals used for fertilization are two of the important issues agriculture is facing in the future. High phosphorus availability in soils is necessary to maintain high agricultural yields. Drought is one of the major threats for terrestrial ecosystem performance and crop production in future. Among the measures proposed to cope with the upcoming challenges of intensifying drought stress and to decrease the need for phosphorus fertilizer application is the fertilization with silica (Si). Here we tested the importance of soil Si fertilization on wheat phosphorus concentration as well as wheat performance during drought at the field scale. Our data clearly showed a higher soil moisture for the Si fertilized plots. This higher soil moisture contributes to a better plant performance in terms of higher photosynthetic activity and later senescence as well as faster stomata responses ensuring higher productivity during drought periods. The plant phosphorus concentration was also higher in Si fertilized compared to control plots. Overall, Si fertilization or management of the soil Si pools seem to be a promising tool to maintain crop production under predicted longer and more serve droughts in the future and reduces phosphorus fertilizer requirements.

## Introduction

Drought is the main constraint in terms of terrestrial ecosystem performance and crop production^1–3^. Drought risks are predicted to increase in future on the continental and the global scale due to climate change^4,5^, threatening agricultural yield and ecosystem performance^6^. Drought-induced loss in crop yield is of global importance for agricultural species selected for their economic yield^7–9^. During prolonged droughts, the soil water storage decreases to values at which water is no longer available for plants, leading to severe drought stress and wilting^10^. During drought, leaf water potential and content decrease substantially, altering transpiration rate and stomatal conductance^11^. Drought effects on crop yields are particularly significant on sandy soils with a low water holding capacity or soils with a high susceptibility for subsoil compaction^12^.

Plants have developed different strategies to cope with water scarcity, such as avoidance of water loss through transpiration, increase of water uptake from the soil by morphological changes in root architecture, or osmotic adjustment^13,14^. There are several measures discussed to adapt or mitigate the upcoming challenges of intensifying droughts. A more recent idea is the fertilization with silicon (Si)^15^.

Si is the second most abundant element in the earth’s crust with approximately 28% (w/w). Primary and secondary minerals as well as biogenic silica can act as a source for different types of silicic acid, as soluble forms of Si^16^. Soluble Si fractions in soils comprise dissolved Si (monosilicic acid, polysilicic acid, and complexes with inorganic compounds)^16^. Plants take up Si either actively or passively in form of monosilicic acid (H_4_SiO_4_), which typically occurs in a range of 0.1–0.6 mM in soil solution^17^, subsequently forming biogenic amorphous silica in the plant tissues^18^. Uptake of H_4_SiO_4_ is mediated by active transport systems allowing the influx into root cells from the soil solution.

On the laboratory scale multiple studies have shown a positive effect of Si fertilization on plant performance under drought conditions. Several parameters were found to be improved after Si-fertilization: higher biomass production^19–22^, larger leaf area^19,23^, elevated root length^20–22^, increased plant nutrition^22^, higher leaf water potential and higher relative plant water content^24^, as well as increase photosynthetic activity and stomatal conductance of drought-stressed plants^20,21,23^.

Most recently, addition of amorphous silica to soils was found to strongly increase the water holding capacity and the plant-available water content of soils^25,26^. An increase of the soil amorphous silica (ASi) by 1% or 5% (weight) increased the plant-available water by up to > 40% or > 60%, respectively and the water content at any water potential^25^. If the water availability in the soil is changed by the addition of Si, soil fertilization with Si may reflect not only physiological responses of drought-stressed plants to Si addition but rather an additive effect of enhanced soil water availability and plant responses to Si accumulation in the substrate. However, little is not known about such effects of ASi-fertilization on soil water availability and plant responses to drought on the field scale. We conducted a plot experiment in the field using different level of ASi-fertilization to examine the effect of soil ASi-fertilization on wheat performance during drought. Our hypotheses were that ASi-fertilization will (1) increase the soil moisture and (2) increase the performance of spring wheat during drought. For this we analyzed soil moisture at different depth, CO_2_ exchange at leaf at plot level, photosynthesis rate and stomatal conductance at the leave level, normalized difference vegetation index, as well as Si concentration in plants and Si availability in soils. As Si is also known to increase plant P concentration^27^ by mobilizing P from soil^28^ we analyzed the P concentration of the wheat plants, too.

## Results

### ASi fertilization increased soil moisture

**Figure 1:**
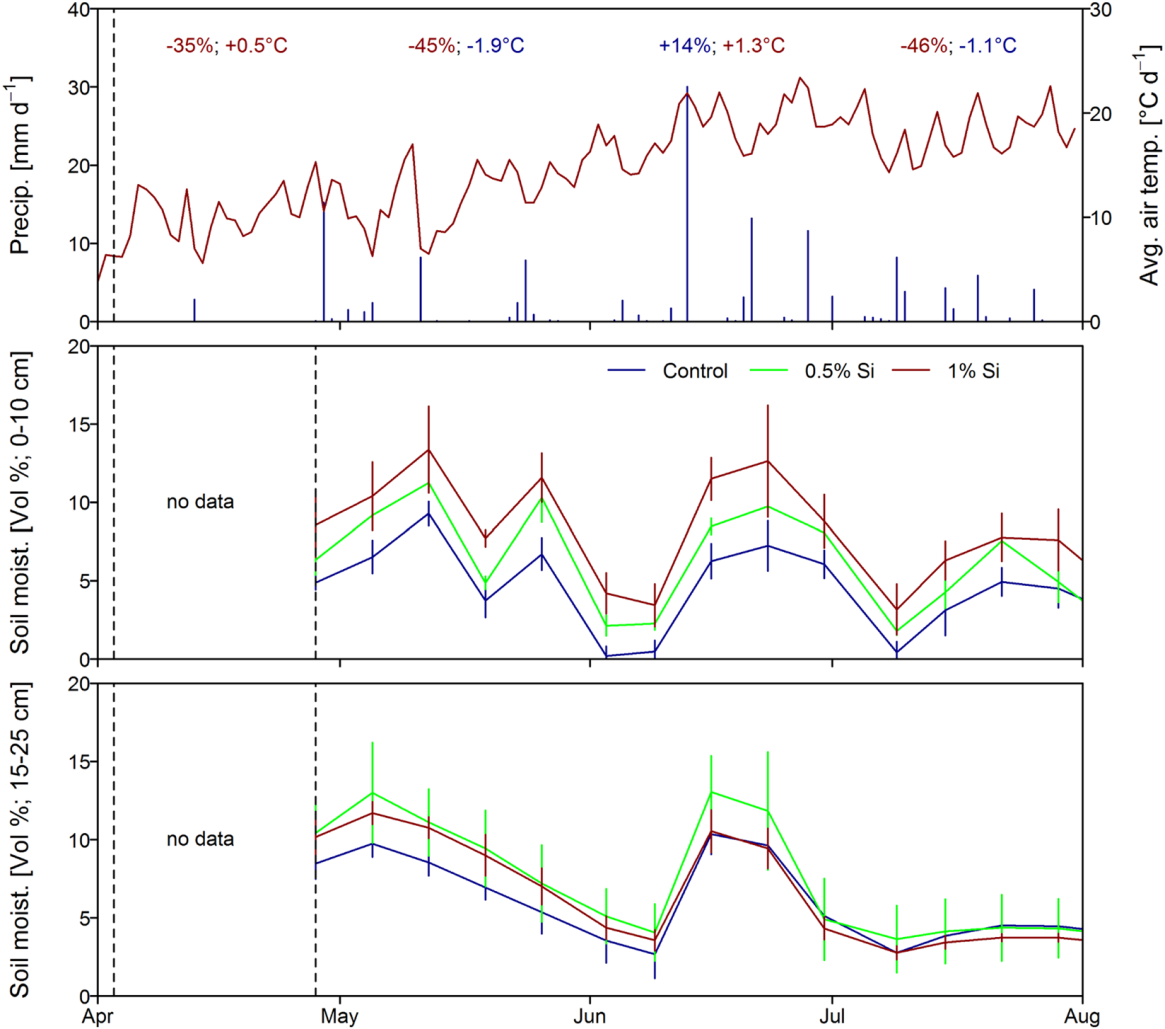
Daily precipitation (mm) and average air temperature (°C) during the crop growth period (top panel). Monthly precipitation and temperature difference (compared to 30 years average monthly precipitation and air temperature; 1990–2020 DWD Station Müncheberg) is given in the top; Corresponding average soil moisture (Vol%) for the uppermost soil layer (0–10 cm; middle panel) and for the depth of 15–25 cm (bottom panel). Error bars indicate the ± 1 SD.

We cultivated summer wheat on a sandy soil in a drought affected region in Germany under three different Si treatments (control, 0.5% and 1% ASi addition into the top 25 cm of the soil) during a rather dry year. From March to May only 68% (March), 45% (April) and 51% (May) of the average rainfall occurred followed by June (96% of average rainfall) which seemingly ended that rather dry period (Fig. 1). Soil moisture of the uppermost soil layer (0–10 cm) was significantly enhanced for the plots with Si addition during the growing season (*p* < 0.001, F = 21.18, df = 2; ANOVA) (Fig. 1). Tuckey post-hoc test revealed significant differences between control and Si0.5% (*p* = 0.007), control and Si1% (*p* < 0.001), and Si0.5% and Si1% (*p* = 0.01). For the soil layer below (10–25 cm) the same trend was found but not significant (*p* = 0.08, F = 2.556, df = 2; ANOVA). Overall, the soil moisture decreased until the heavy precipitation event in the middle of June (Fig. 1).

### Higher NDVI for ASi fertilized plots

**Figure 2:**
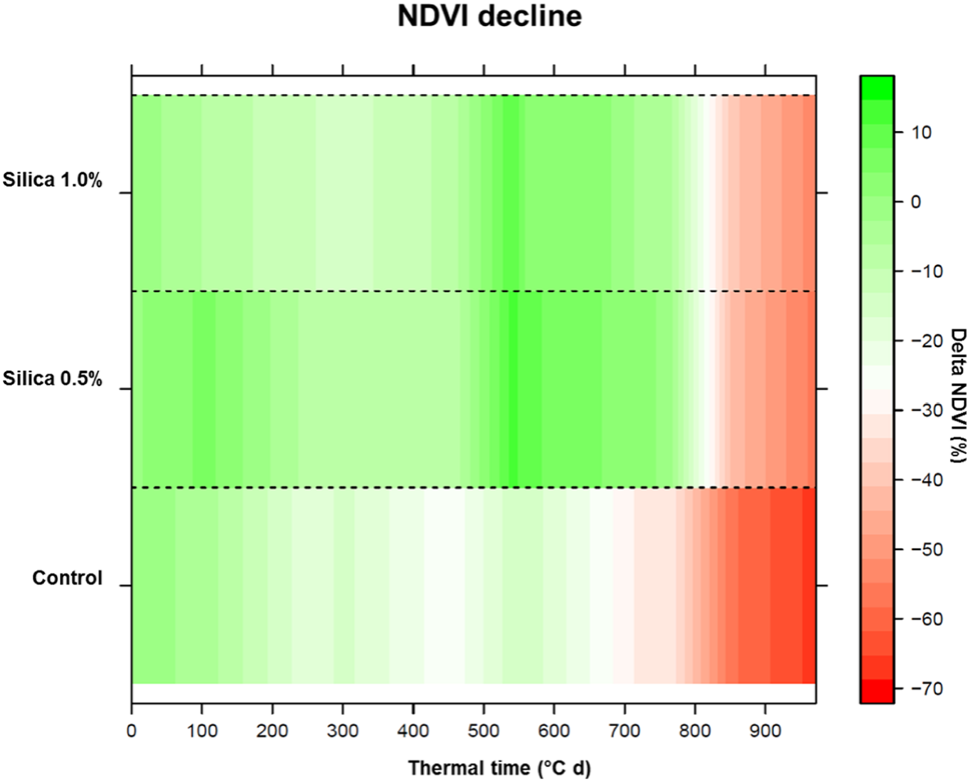
Heatmap plot of average measured NDVI decline (in %; based on average initial NDVI measurement; linear interpolated) over thermal time (°C d) for the different treatments (control, 0.5% ASi and 1% ASi).

Control plots showed a slightly higher normalized difference vegetation index (NDVI) at the start of the observation period compared to the Si fertilized plots. Starting mid of June till the end of the experiment, however, the Si fertilized plots evidenced a substantially higher NDVI. In addition, NDVI for the Si fertilized plots remained higher for a longer period of time (Fig. 2). This trend was significant (*p* < 0.001, F = 170.5, df = 2; ANOVA). In general, NDVI followed the pattern of soil moisture with a decrease during the observation period but temporary increase after a heavy precipitation event during mid of June.

### Strong effects of Si on R_eco_, GPP and net photosythesis but not NEE

**Figure 3:**
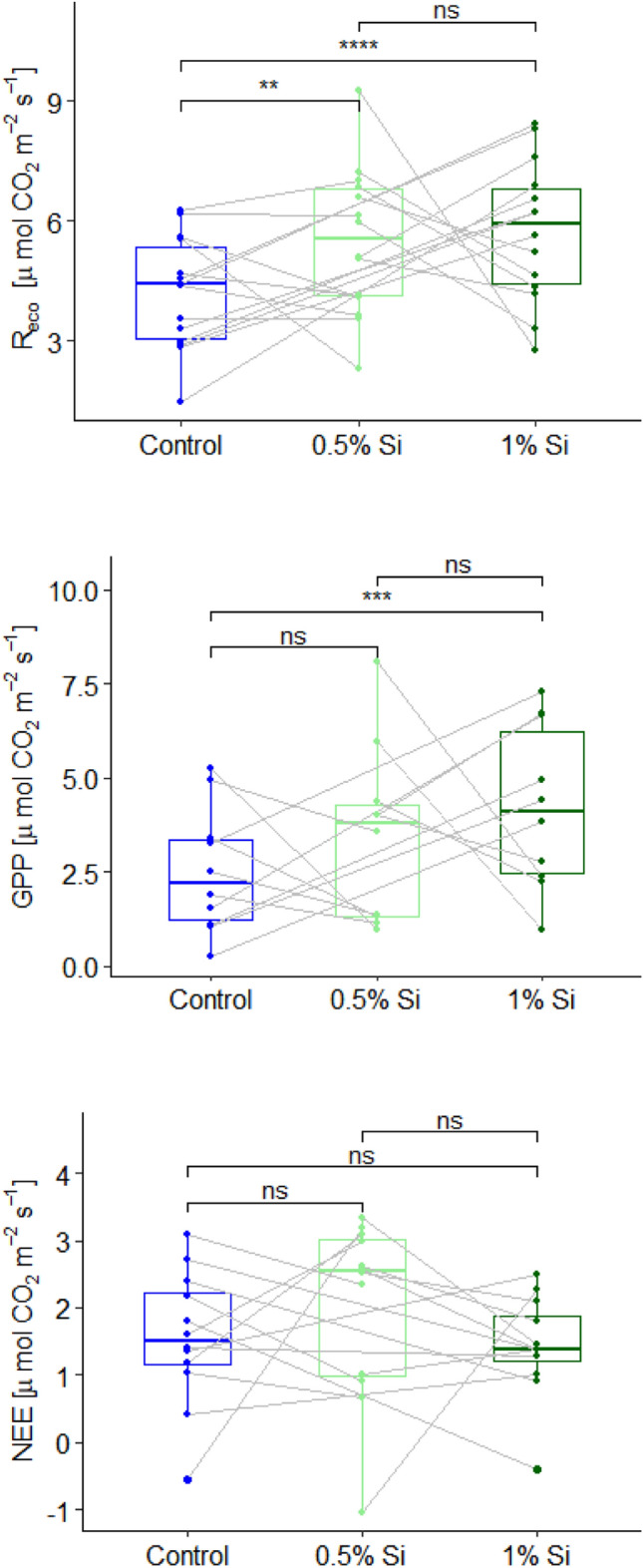
Closed chamber based R_eco_, GPP and NEE fluxes grouped for the different treatments (control, 0.5% ASi and 1% ASi) and pairwise compared (lines show data pairs of the different measurement campaigns).

CO_2_ gas exchange measurements (closed chamber measurements) were significantly higher for R_eco_ for both Si fertilized treatments (28% (*p* = 0.001; paired *t*-test) for the Si0.5% and 43% (*p* < 0.001; paired *t*-test) for the Si1%; paired *t*-test) compared to the control (Fig. 3). No significant differences (*p* = 0.57; paired *t*-test) were found between Si0.5% and Si1% for R_eco_. The mean GPP flux of the Si0.5%-treatment was 34% and of the Si1% was 71% higher compared to the control (Fig. S2). GPP was only significant different (*p* < 0.001; paired *t*-test) between Si1% and control (Fig. 3). No significant differences (*p* = 0.248; paired *t*-test) were found between Si0.5% and Si1% as well as between Si0.5% and control (*p* = 0.201; paired *t*-test) for GPP. No significant differences were found for net ecosystem exchange (NEE) between all treatments (*p* = 0.255 for control vs. Si0.5%; *p* = 0.521 for control vs. Si1%; and *p* = 0.179 for Si0.5% vs. Si1%; all paired *t*-test).

**Figure 4:**
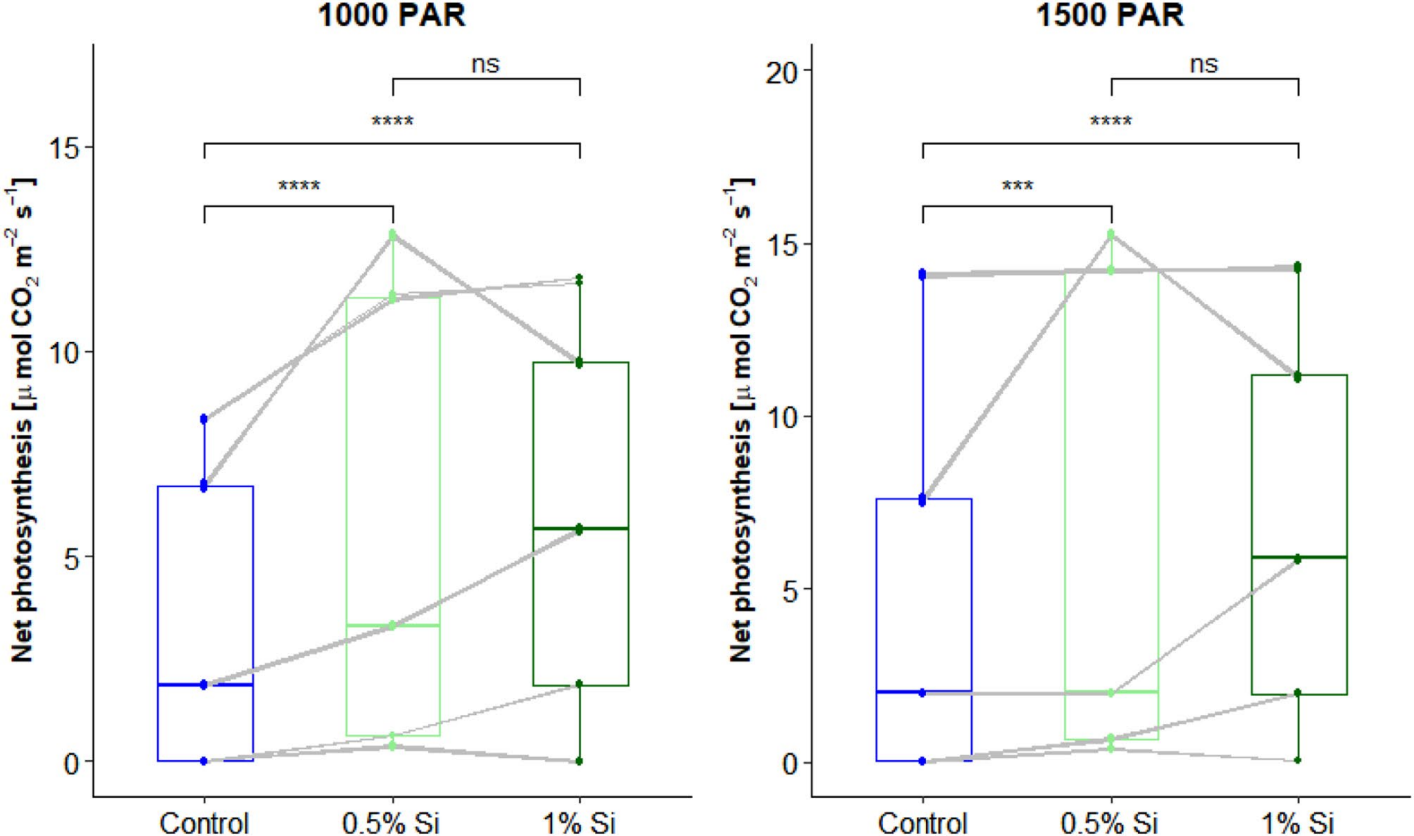
Measured leaf level net photosynthesis for the different treatments (control, 0.5% ASi and 1% ASi) at different light intensity levels. Significance level **** equals *p* < 0.001. Lines show data pairs each from one measurement date.

CO_2_ gas exchange measurements at the leaf level (GFS-3000-measurements) showed a significant (*p* < 0.001 paired *t*-test) higher net photosynthesis for both Si fertilization treatments (Si0.5% and Si1%) compared to the control for light intensities of 1000 and 1500 PAR, respectively (Fig. 4).

Hence both, ecosystem and leaf level CO_2_ gas exchange measurements showed higher CO_2_ uptake of the Si fertilized treatments compared to the control.

### Faster reaction of Si-fertilized plants to light intensity changes

**Figure 5:**
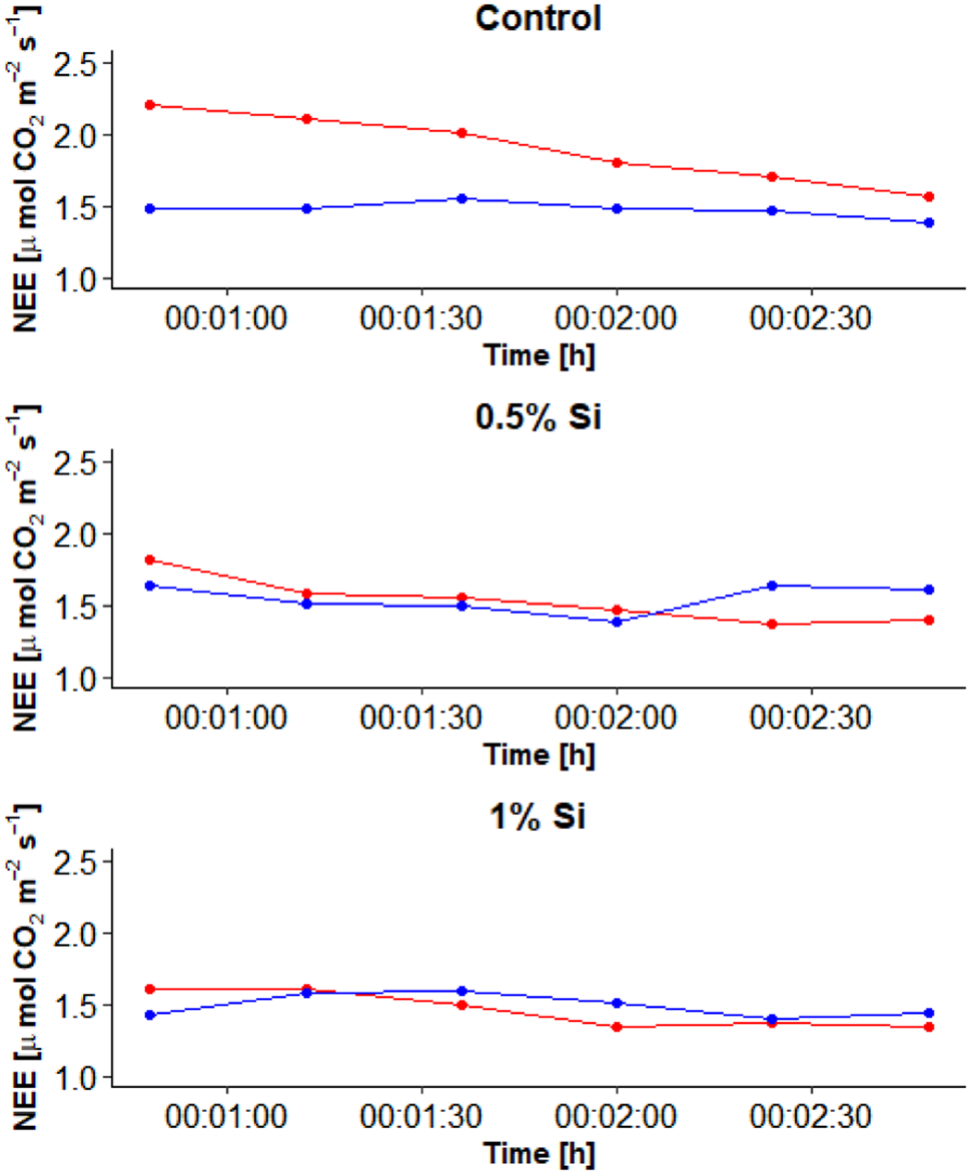
Net ecosystem exchange (NEE) data for the different treatments (control, 0.5% ASi and 1% ASi) at different time points prior (blue) and after shading (red). Data from chamber measurements.

Chamber measurements of NEE prior and after shading revealed a much faster response following shading for both Si-fertilized treatments (Fig. 5). In case of the control, the NEE fluxes prior shading were more or less constant at 1.5 µmol CO_2_ m^−2^ s^−1^ whereas after shading the NEE was higher (2.2 µmol CO_2_ m^−2^ s^−1^) and decreased only over time back to its initial (prior shading) value (1.6 µmol CO_2_ m^−2^ s^−1^). Compared to that, the NEE for both Si treatments was prior and after shading nearly constant (~ 1.5 µmol CO_2_ m^−2^ s^−1^). For the control treatment, the higher NEE after shading suggests a lower GPP after shading due to the slower light adaptation of the control treatment compared to both Si-fertilized treatments (Fig. S3).

**Figure 6:**
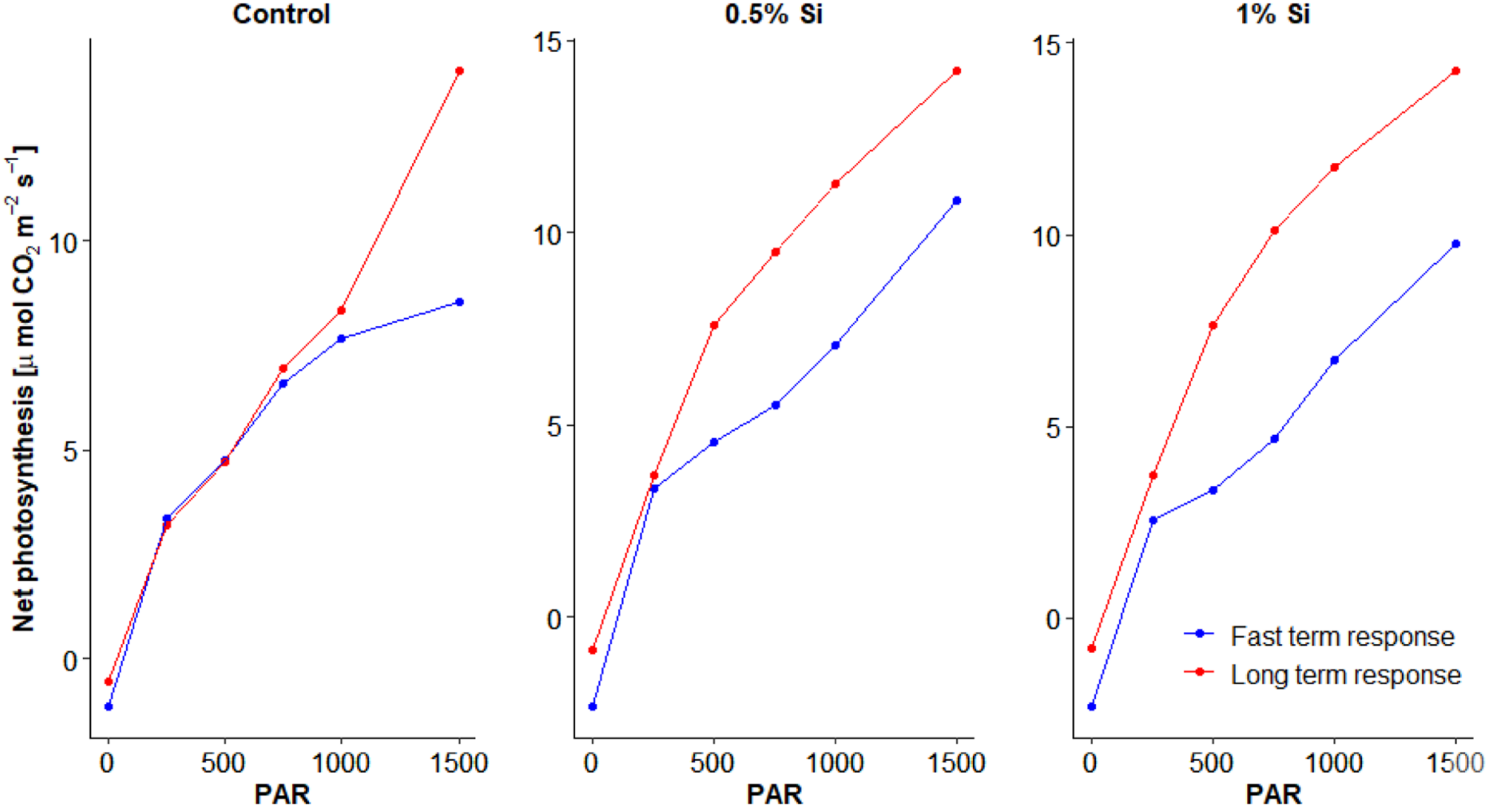
Fast and long term measurements of net photosythesis for the different treatments (control, 0.5% ASi and 1% ASi) at different light intensity. Data from GFS-3000 measurements at the leaf level.

Net photosynthesis measurements on leaf level with short and longer term adaptation time after light intensity changes did not differ for control, but Si fertilized treatments. Thus, a saturation of net photosynthesis with higher PAR was neither obtained for the long nor short adaptation time of the control, but for the longer term adaptation time for both Si fertilized treatments (Fig. 6). This suggests a lower light adaptation of the control treatment compared to both Si-fertilized treatments (Fig. 6). CO_2_ gas exchange measurements at the leaf level (GFS-3000) were thus in line with results of a faster reaction of Si fertilized plants to light intensity changes obtain by closed chamber measurements on ecosystem level.

### Si fertilization increased the wheat P and Si concentration

**Figure 7:**
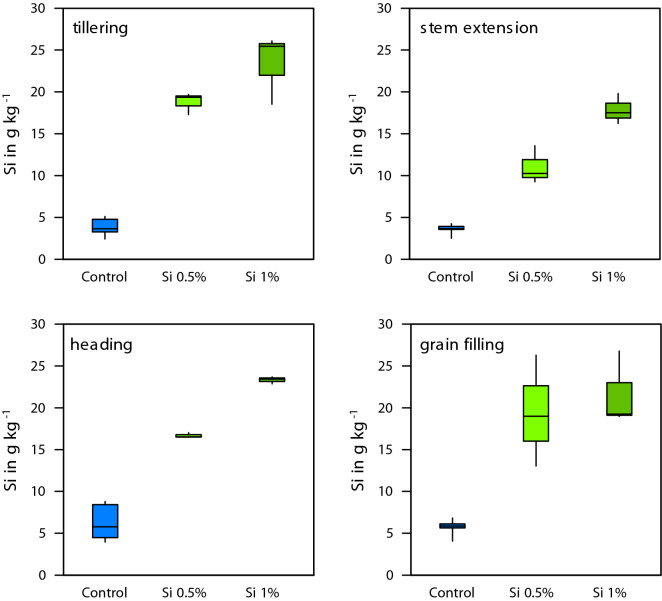
Leaf silicon concentration for the different treatments over the growing season.

**Figure 8:**
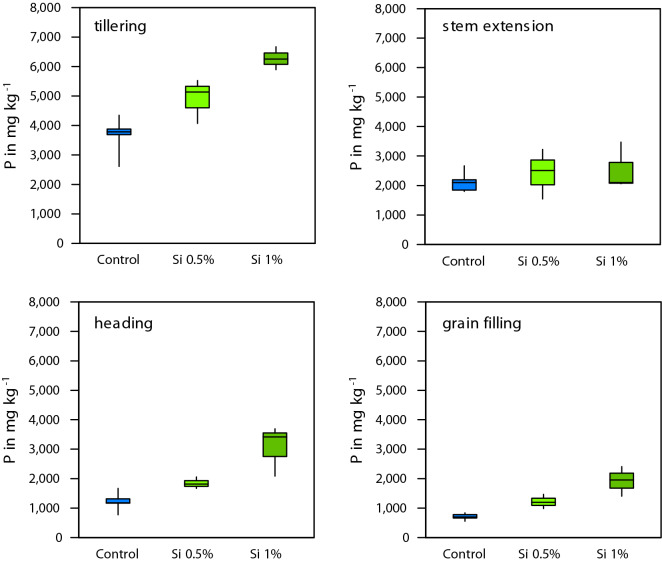
Leaf phosphorus concentration for the different treatments over the growing season.

Si fertilization increased Si content in leaves at tillering stage (*p* < 0.001; F = 95.256, df = 2, ANOVA), stem extension (*p* < 0.001; F = 102.815, df = 2, ANOVA), heading (*p* < 0.001; F = 128.553, df = 2, ANOVA), and grain filling (*p* < 0.001; F = 22.852, df = 2, ANOVA) (Fig. 7). Additionally, Si fertilization increased P content in leaves at tillering (*p* < 0.001; F = 20.071, df = 2, ANOVA), heading (*p* < 0.001; F = 15.647, df = 2, ANOVA), and grain filling (*p* < 0.001; F = 19.889, df = 2, ANOVA), but was not significantly different at stem extension stage (*p* = 0.574; F = 0.591, df = 2, ANOVA) (Fig. 8).

### Higher soil Si availability after Si fertilization but no effect on soil P

The CaCl_2_ extractable Si and the alkaline extractable ASi of the different plot was not different (*p* = 0.655, F = 0.444, df = 2 for CaCl_2_ extractable Si and *p* = 0.833, F = 0.186, df = 2 for ASi, both ANOVA) before Si fertilization. The plots had a CaCl_2_ extractable Si content for control of 4.8 ± 0.6 mg kg^−1^ for Si0.5% of 5.4 ± 1.4 mg kg^−1^, and for Si1% of 5.0 ± 0.7 mg kg^−1^ and ASi concentrations of 0.4 ± 0.1 g kg^−1^ (control), 0.38 ± 0.1 mg kg^−1^ (Si0.5%) and 0.35 ± 0.1 mg kg^−1^ (Si1%). After Si fertilization, we found significant higher CaCl_2_ extractable Si for the Si fertilized plots (*p* < 0.001, F = 121.3, df = 2, ANOVA) and for ASi (*p* < 0.001, F = 393.2, df = 2, ANOVA) at tillering stage (Fig. S4). At grain filling, we found significant higher CaCl_2_ extractable Si for the Si fertilized plots (*p* < 0.001, F = 336.4, df = 2, ANOVA) and for ASi (*p* < 0.001, F = 145.0, df = 2, ANOVA) (Fig. S4). We found no differences (*p* = 0.413, F = 0.978, df = 2, ANOVA) of soil P_cal_ between the different treatments (control 49.2 ± 4.9 mg kg^−1^; Si0.5% 51.8 ± 3.0 mg kg^−1^; Si1% 47.3 ± 1.3 mg kg^−1^) at tillering stage.

## Discussion

### Better plant performance during drought after soil ASi fertilization

Our results clearly show an improved performance of wheat during drought after ASi fertilization. It is obvious that the increased moisture of the ASi fertilized soils contributes to better performance of wheat under drought.

It was already shown in a lysimeter study and by HYPROP™ analysis that soils fertilized with ASi have a higher water holding capacity and more plant available water^25,26^. However, the present study showed for the first time a positive effect of ASi fertilization on soil moisture at the field scale. Improved soil moisture and increasing water availability is essential for plants to maintain their physiological processes during dry periods.

If water shortages persists for a longer time, the plant will start senescence^29^, which accelerates in the presence of biotic or abiotic stresses such as drought^30^. A delayed leaf senescence is thus a measure for higher drought tolerance of plants^31^. Senescence is typically characterized by a loss in chlorophyll^32^ and a subsequent decline in photosynthetic activity^33^. In the present study, a later growth decline of Si fertilized plants compared to control was found. This is indicated by a later decline in measured NDVI (Fig. 2) and an in general higher leaf and ecosystem level CO_2_ gas exchange (i.e., photosynthetic activity; Figs. 3 and 4), especially after the heavy precipitation event at 13th of June. NDVI varied between 0.6 and 0.2. This is within the lower end of the range of NDVI during wheat growth cycle presented by e.g., Hassan et al. (2019) and comparable to values given for NDVI measurements on wheat during grain-filling by e.g., Lopes and Reynolds^34^. A decline in net canopy photosynthesis of wheat with senescence was inter-alia shown by Rodriguez et al.^35^. On ecosystem level e.g., Moureaux et al.^36^ presented a decline of GPP and R_eco_ [due to autotrophic respiration (R_a_)] for winter wheat following crop senescence. The fact that no significant difference in NEE was found in the present study between the control and Si fertilized plants (Fig. 3), can be explained by a decline in both, above- and belowground R_a_ with progressing senescence. Thus, the net canopy photosynthesis might be significantly different but the overall NEE (including belowground autotrophic and heterotrophic respiration) is not different.

Hence, we argue that Si fertilized plants seem to be less affected by drought enabling the plants to start senescence later compared to plants from the control plots (not fertilized with ASi), which suffer more from water limitation, and being already in a progressed stage of senescence, i.e. lower NDVI and photosynthetic activity. Thus, those plants from the control treatment may not respond to the increase in soil water availability following the heavy rainfall event to a similar extent as plants in the plots fertilized with ASi.

On the contrary plants from the ASi fertilized plots maintained higher physiological activity and did not progress into senescence early (higher NDVI and gas exchange compared to the plants from the control plots) during the drought period until the 15th of June. This is likely because of the higher soil moisture in these plots enabling those plants from ASi fertilized plots to increase the photosynthesis rate stronger after the heavy rainfall. This higher photosynthesis rate of the plants from the ASi fertilized plots seem to maintain until the end of July, although on a lower level due to the proceeding drought decreasing plant water availability by reducing soil moisture. NDVI followed the pattern of soil moisture with a general decrease during the observation period, indicating crop senescence. In addition, NDVI was in general rather low indicating the negative effect of the drought period.

Our results are in line with other studies clearly showing that Si addition leads to increased photosynthetic rate and also stomatal conductance under drought^20–22,37^. The faster reaction of stomatal conductance to changed light intensity of Si fertilized compared to unfertilized (control) plants as found in our study is in line with other studies^38^ and may be explained by a more progressed senescence of the control plants, as indicated by NDVI (Fig. 2). The GFS-3000 measurements at the leaf are highly heterogenic because of differences in leaves and between certain plants, but the data obtain by this technique shows the same trend as data from closed chamber measurements. Si is known to protect plants from drought stress via different mechanisms^15^. For example Si may promote growth of roots and with this may improve plant water uptake by penetrating a larger volume of soil by the root and maintaining high cell turgor^39^. It was further suggested that Si deposition in the endodermal cell wall may improve water uptake^40^. Silicon in stomata cells may be involved in stomatal movement and conductance potentially reducing water loss through stomata^37,41^. The Si-double layer was found to be able to reduce water loss from the cuticle by more than 20%^42^. Water loss via stomata is the most important process for water losses during drought^41,43^. However, a more recent study indicated that the positive effect of Si on plant performance during drought is rather due to the increase of soil water availability upon soil Si addition and less due to plant Si accumulation^44^. This is in line with finding of the present study also showing a strong interdependency between soil moisture increase after soil Si addition and plant performance during drought.

### Increased wheat P and Si concentration after Si fertilization

The enhanced P concentration after Si fertilization is in line with previous lab studies, but this time shown at the field scale^27^. The increase in the plant P status can be explained by potential silicic acid mobilization from the Si-fertilizer. Silicic acid is mobilized as polysilicic acid which has a high binding affinity to the surfaces of soil particle outcompeting P^16,26,28^. With this silicic acid is able to mobilize P from the soil pool of unavailable P making this P available for plant uptake consequently increasing the plant P concentration^27,28^. Hence, Si fertilization is a tool to reduce the need of agriculture for P-fertilizer by mobilizing P from unavailable pools in the soil. However, despite higher P concentration in the plants, soil P availability in calcium acetate-lactate extracts (P_cal_) did not differ between the control and Si treatments at tillering stage.

The higher availability of Si after Si fertilization also led to a strong increase of the plant Si status, which is in line with earlier studies using the same Si fertilizer^27,45^. The increased plant Si status after Si fertilization indicated also that the unfertilized soils had a low Si availability. The increased plant Si concentration in the Si fertilized plots can be explained by the higher Si availability (both CaCl_2_ and alkaline extracted) after Si fertilization.

## Conclusion

Overall, the data from the present study clearly showed that soil ASi fertilization can be used as measure to improve crop plant performance during drought. This improved plant performance is ensured by increasing soil moisture and with this improved soil water availability for plants. This higher soil water availability for plants and potentially the ability of the plants to reduce water losses by faster stomata responses leads to later induction of irreversible plant senescence which ensures higher productivity during drought periods. Another important factor potentially improving the crop plant performance during drought is the increased P concentration of plants in ASi fertilized plots as Si is able to mobilize P from plant unavailable pools in soils improving the plant P concentration. Hence, ASi fertilization seems to be an effective tool to mitigate drought stress and increase plant P concentration. Consequently, the management of soil Si pools is highly important to maintain crop production under the predicted longer and more serve drought periods in future.

## Methods

### Study site and experimental design

Our study site is located at ZALF’s experimental area near Müncheberg, NE Germany (52.5176° N, 14.1300° E). The climate is characterized by a mean annual precipitation of 555 mm and a mean annual temperature of 8.9 °C (ZALF weather station, 1981–2010). The soils have been developed from aeolian and glaciofluvial sands overlying a thin layer of glacial till. Due to the strongly variable, upper boundary of the clay-enriched Bt horizon (80–120 cm) soils classifies either as Albic Luvisol (Arenic, Aric, Neocambic) or as Albic, Lamellic Arenosol (Aric)^46^. The soil texture of the at least upper 80 cm is dominated by medium and fine sand with intercalated clay lamellae of 2–4 cm thickness. By soil augering we determined a high soil heterogeneity—mainly due to the variable depth of the Bt horizon (sandy loam) and the presence/absence of small clay lamellae. As these horizons not only store more plant-available water and nutrients, but reduce seepage (lower hydraulic conductivity), their local upper boundary determine local rooting space depth/quality, hence local crop growth conditions, especially during dry spells.

The experimental area of our study captures 18 × 14 m^2^ (Figure S1). The characterization of the soil can be found in Table S1. The treatments were conducted at single plots 3 × 4 m^2^. Six plots form control (without Si addition), three plots received 0.5%ASi (Aerosil 300, Evonik Industries, Germany) and three plots 1%ASi in the top 25 cm of the soil. We used a block design for practical reason with distance plots (buffer stripes) between Si fertilized and control plots with the same size as all other plots to avoid interferences between the treatments. The ASi was mixed in four weeks prior sowing both by hand and with the cultivator. The field cultivator was used in the same way for the control plots to ensure the same physical disturbance of all treatments. Afterwards, all plots were irrigated with 60 mm m^−2^ using an overhead sprinkling system to enable similar soil moisture for all plots.

Summer wheat (*Triticum aestivum* L., cv. *Tybalt*) was sown (417 grain per m2) in all plots using a sowing machine. The seeds were purchased from Saaten-Union GmbH (Germany). D. Barkusky identified the plant material in our study. The permission to cultivate and collect the plants from the experimental site was ensured by the land owner, which is our research institute (Leibniz Centre for Agricultural Landscape Research; ZALF). Fertilization with nitrogen (N) and sulfur was done at tillering (50 kg ha^−1^ a^−1^ for nitrogen and 12 kg ha^−1^ a^−1^ for sulfur) and at heading stage (90 kg ha^−1^ a^−1^). To protect the plants against herbivory all plots were treated with Wildgranix (SeNaPro GmbH, Germany) (200 kg ha^−1^) at tillering and against weeds with Ariane ™ C (1.5 L ha^−1^) at stem extension stage.

### Sampling and measurements

#### Sampling and analyses

At four different growing stages (tillering, stem extension, heading, and grain filling) we collected five plants from each treatment and analyzed a mixed sample of the leaves of the five plants for Si and phosphorus (P). For this the leaves were dried, ground and extracted. Si was extracted from 0.03 g shoot material with 30 ml Na_2_CO_3_ at 85 °C and filtrated at pore-size of 0.2 mm. For P, 0.1–0.2 g shoot material was digested in a microwave digestion system (CEM-Mars5, CEM Corporation, Matthews, NC, USA) at 180 °C with 3 ml HNO_3_ and 2 ml H_2_O_2_. The soil prior and after Si fertilization (tillering and grain filling stage) were analysed for CaCl_2_ extractable Si using 0.01 M CaCl_2_ for 1 h to extract soluble Si and 0.1 M NaCO_3_ to extract alkaline extractable amorphous Si (ASi)^16^. Soil P availability was determined at tillering stage using calcium-acetate-lactate extract (P_cal_) according to Schüller^47^. Si and P concentrations in all extracts were measured at an ICP-OES (Varian, Vista-Pro radial, Palo Alto, California, USA).

Soil moisture at 0–10 cm and 15–25 cm depth was analysed biweekly at all treatments using TDR-sensors (FP/mts equipped to a FOM/mts, E-Test Sp. z o.o., Poland).

#### Leaf level CO_2_*exchange measurements*

The net CO_2_ assimilation rate (net photosynthesis) of leaf level was obtained every two weeks from June to July 2020 using a gas-exchange measurement device (GFS-3000, WALZ, Germany). The stomatal conductance and net CO_2_ assimilation rate were measured for 5 min in PAR steps of 250 μmol m^−2^ s^−1^ ranging from 0 to 1500 μmol m^−2^ s^−1^ with a short (5 min) and longer adaptation time (20 min) to set light intensity gradients.

On each measurement day, the net CO_2_ assimilation rate of 9 leaves (three per treatment) was measured. Measurements were performed under conditions of constant air temperature (27 °C) and relative humidity (75%) within the measurement cuvette. After the measurements, leaves were clipped and the respective leaf area was determined.

#### Ecosystem level CO_2_ exchange measurements

CO_2_ exchange measurements (NEE and R_eco_) were conducted between 10:00 and 14:00 every two to three weeks during June and July 2020 using a flow-through non-steady-state (FT-NSS) manual closed chamber system in a dynamic mode^48,49^. The transparent (76% light transmission; NEE flux measurements) and opaque (ecosystem respiration (R_eco_) flux measurements) cone shaped PVC chambers had a flat top and total volume of 0.0672 m^3^ and were equipped with a fan for efficient headspace mixing. For measurements, the chamber was mounted on pre-installed frames^50^. CO_2_ concentration changes during chamber deployment were recorded using an infrared gas analyzer (LI-850, LI-COR Biosciences, USA) combined with a membrane pump and connected to a CR1000 data logger (Campbell Scientific Ltd., USA).

On each measurement day, NEE and R_eco_ fluxes were measured by alternately deploying the opaque and transparent chambers on all plots. During the individual 4 min measurements, CO_2_ concentration changes in the chamber headspace were determined at a 3-s interval. During the measurement, air temperature inside and outside the chamber, soil temperatures and PAR (inside the chamber) were recorded.

#### Flux calculation and separation of NEE into GPP and Reco

Prior to NEE (transparent chamber measurements) and R_eco_ (opaque chamber measurements) flux calculation a death-band of 10% (exclusion of first and last 10% of data records) was applied to the data of each chamber measurement. Thus, data noise that originated from either turbulence or pressure fluctuation caused by chamber deployment was excluded^51–53^. In addition, a water vapor correction of measured CO_2_ concentrations (CO_2c_) was performed (Eq. S1; Webb, et al. ^54^.

S1 $$ CO_{2c} = CO_{2s} \cdot \frac{{1 - H_{2} O_{r} /1000}}{{1 - H_{2} O_{s} /1000}} $$

where CO_2s_ and H_2_O_s_ denote the respective measured CO_2_ and H_2_O concentration, while H_2_O_r_ denotes the initially recorded H_2_O concentration (reference) of a chamber measurement. Measured CO_2_ exchanges (R_eco_ fluxes (opaque chamber) and NEE fluxes (transparent chamber) in g C m^−2^ s^−2^) were calculated based on the ideal gas law using a linear regression approach (Eq. S2):

S2 $$ {\text{f}} = \frac{{{\mathrm{MpV}}}}{{{\mathrm{RTA}}}} \cdot \frac{{{\Delta c}}}{{{\Delta t}}} $$

where M denote the molar mass of the gas (g mol^−1^), p denote the ambient air pressure (Pa) and V denote the chamber volume (m^3^). Since plants accounted for < 0.1% of the total chamber volume, a static chamber volume was assumed. R denote the gas constant (8.314 m^3^ Pa K^−1^ mol^−1^), T denote temperature inside the chamber (K), A denotes the basal area (m^−2^) and Δc/Δt denotes the linear CO_2_ concentration change over time (estimated using the least squares method; e.g., Leiber-Sauheitl et al.^55^). The variables K as well as more importantly dc/dt, were obtained by applying a variable moving window (min. length 30 s) to each chamber measurement, which enables multiple data subsets per chamber measurement. Thus, multiple fluxes were calculated per chamber measurement. These resulting CO_2_ fluxes per measurement (based on the moving window data subsets) were further evaluated according to the following exclusion criteria: (1) fulfilled prerequisites for applying a linear regression (normality (Lillifor´s adaption of the Kolmogorov–Smirnov test), homoscedasticity (Breusch-Pagan test) and linearity); (2) significant regression slope (*p* ≤ 0.1, *t*-test); (3) range of within-chamber air temperature not larger than ± 1.5 K (R_eco_ and NEE fluxes) and a PAR deviation (NEE fluxes only) not larger than ± 20% of the average to ensure stable environmental conditions within the chamber throughout the respective measurement; (4) no outliers present (± 6xIQR). Calculated CO_2_ fluxes that did not meet all exclusion criteria are discarded. In cases where more than one flux per measurement met all exclusion criteria, the CO_2_ flux with the steepest slope was chosen. Since GPP cannot be measured directly, GPP fluxes were obtained indirectly by addition of measured R_eco_ fluxes to measured NEE^48^.

#### NDVI measurements

Normalized difference vegetation index (NDVI) was used to determine and follow senescence^32,34,56^. NDVI was chosen as a good indicator of canopy greenness and photosynthetic size^32^, being highly responsive to the loss of chlorophyll and browning of crops, which accompanies senescence^57^. Measurements were performed weekly starting from stem extension for each of the plots using a near-infrared (NIR)/visible light (VIS) double, 2 canal sensor device (SKR 1850, Skye Instruments Ltd., UK) mounted on a 1.8 m handheld pole^58,59^, connected to a CR1000 data logger (Campbell Scientific Ltd., USA). The double, 2 canal sensor device consist of an up- and downward sensor, measuring the incoming (VISi) and reflected (VISr) VIS at a wavelength of 656 ± 10 nm and incoming (NIRi) and reflected (NIRr) NIR at 780 ± 10 nm. The upward sensor was fitted with a cosine-correction diffusor for measurements of the incident radiation, while the downward sensor had a 25° cone field of view, thus covering an area of 0.5 m^2^ during measurements. For each plot, 3 successive 10 s (= 10 records) measurements were performed. NDVI was calculated as NDVI = ((NIRr/NIRi)-(VISr/VISi))/ ((NIRr/NIRi) + (VISr/VISi)).

#### Statistics

The obtained data were statistically analysed using IBM® SPSS® Statistics (Version 21.0, IBM, Armonk, NY, USA). An ANOVA with Tuckey post-hoc test or a paired *t*-test were carried out to prove statistical differences between different treatments.

## Supplementary Information

Supplementary Information.
