# Supplementary material for: Silica fertilization improved wheat performance and increased phosphorus concentrations during drought at the field scale
Source: Sci Rep. 2021 Oct 21;11:20852. doi: 10.1038/s41598-021-00464-7 (PMC8531131; doi:10.1038/s41598-021-00464-7)
Supplement: Supplementary file 1 — Supplementary Information. [file 41598_2021_464_MOESM1_ESM.docx]

**Supplementary material to:**

**Silica fertilization improved wheat performance and increased phosphorus concentrations during drought at the field scale**

Running title: Silica improves drought performance of wheat

Jörg Schaller ^1, *^, Eric Scherwietes ^2^, Lukas Gerber ^2^, Shrijana Vaidya ^3^, Danuta Kaczorek ^4^, Johanna Pausch ^2^, Dietmar Barkusky ^5^, Michael Sommer ^1,6^, and Mathias Hoffmann ^3^

^1^ “Silicon biogeochemistry” working group, Leibniz Centre for Agricultural Landscape Research (ZALF), 15374 Müncheberg, Germany.

^2^ Agroecology, University of Bayreuth, 95440 Bayreuth, Germany.

^3^ “Isotope Biogeochemistry and Gas Fluxes” working group, Leibniz Centre for Agricultural Landscape Research (ZALF), 15374 Müncheberg, Germany.

^4^ “Landscape Pedology” working group, Leibniz Centre for Agricultural Landscape Research (ZALF), 15374 Müncheberg, Germany.

^5 “^Experimental Infrastructure Platform”, Leibniz Centre for Agricultural Landscape Research (ZALF), 15374 Müncheberg, Germany.

^6^ University of Potsdam, Institute of Geography and Environmental Science, 14476 Potsdam, Germany.

^*^ Corresponding author: telephone: +49 33432 82137; Email address: [joerg.schaller@zalf.de](mailto:joerg.schaller@zalf.de)

**TABLE S1**. Soil properties of an Albic Luvisol (Arenic, Aric, Neocambic) 50 m NNW of the experimental plot.

| **Horizon (WRB)** | **depth (cm)** | **% sand*** | **% silt** | **% clay** | **pH (KCl)** | **Fe_o_ (g kg^-1^)** | **Fe_d_ (g kg^-1^)** | **Fe_o_/Fe_d_** | **Total-P (g kg^-1^)** | **P_CAL_* (mg kg^-1^)** |
| --- | --- | --- | --- | --- | --- | --- | --- | --- | --- | --- |
| Ap1 | 0-25 | 74 | 21 | 5 | 5.3 | 0.22 | 0.30 | 0.73 | 0.50 | 58 |
| Ap2 | 25-35 | 73 | 21 | 6 | 5.0 | 0.15 | 0.28 | 0.54 | 0.42 | 44 |
| Bw | 35-45 | 75 | 22 | 3 | 5.3 | 0.07 | 0.21 | 0.33 | 0.25 | 18 |
| II E | -75/100 | 86 | 11 | 3 | 5.6 | 0.06 | 0.17 | 0.35 | 0.18 | 9 |
| Bt | -95/120 | 74 | 11 | 15 | 5.7 | 0.18 | 0.73 | 0.25 | 0.43 | 18 |
| III Ck | -135/160 | 40 | 34 | 26 | 6.8 | 0.13 | 0.69 | 0.19 | 0.55 | 18 |
|  |  | *mainly fine and medium sand |  |  |  |  |  |  |  | * plant-available P |

**
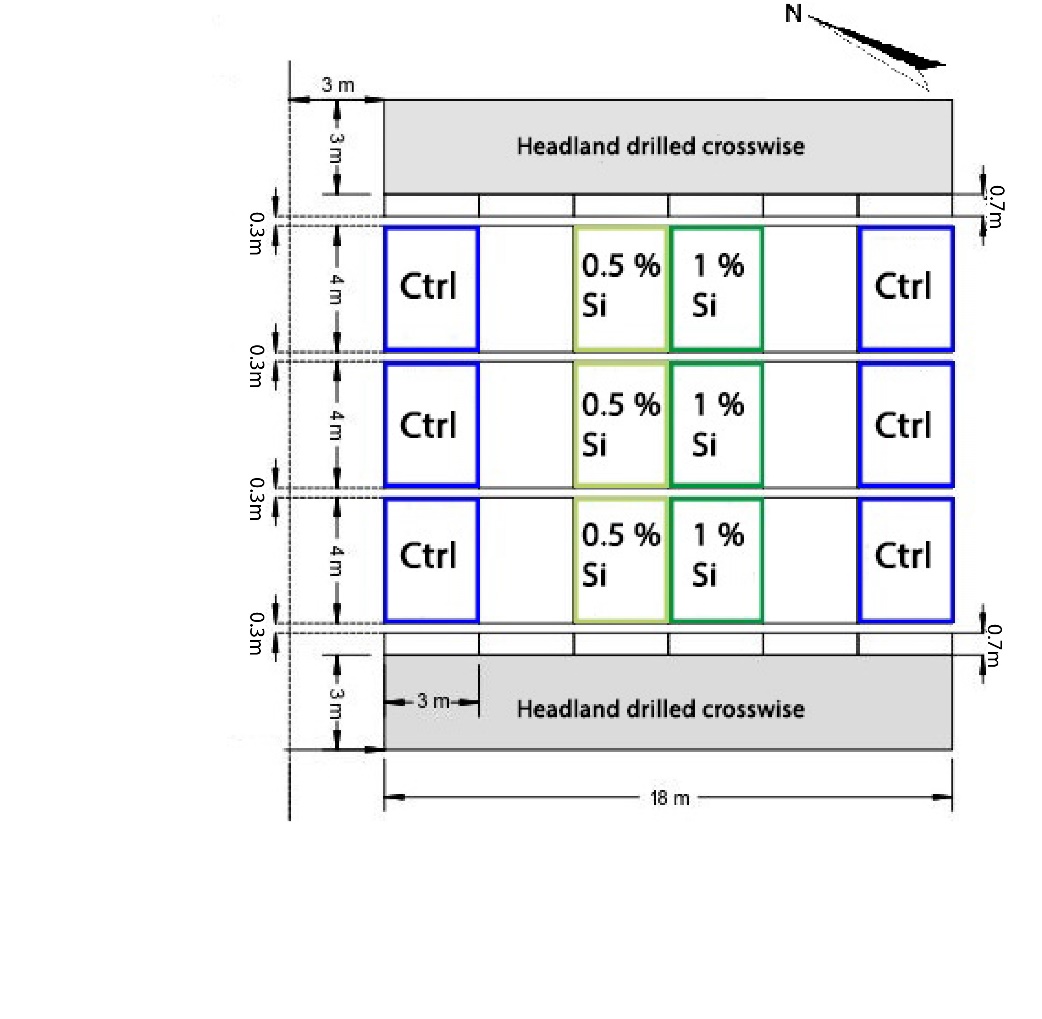
**

**FIGURE S1 Experimental design showing the different treatments (control, 0.5% ASi addition (0.5%Si), and 1%ASi addition (1%Si), with their replicates at the field site.**


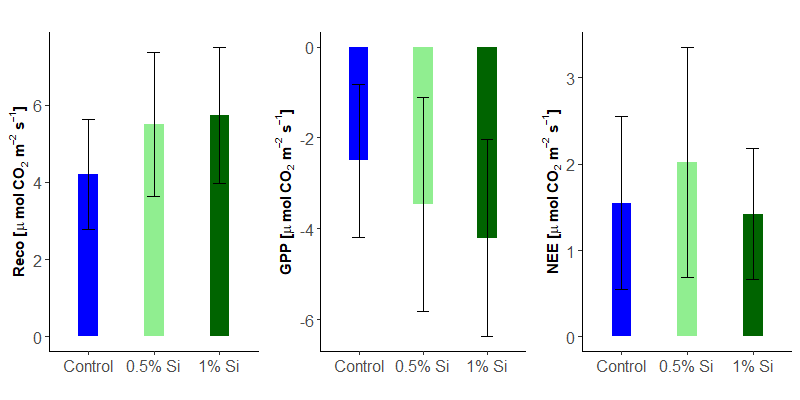


**FIGURE S2: Mean R_eco_, GPP and NEE of the different treatments (control, 0.5% ASi and 1% ASi).**


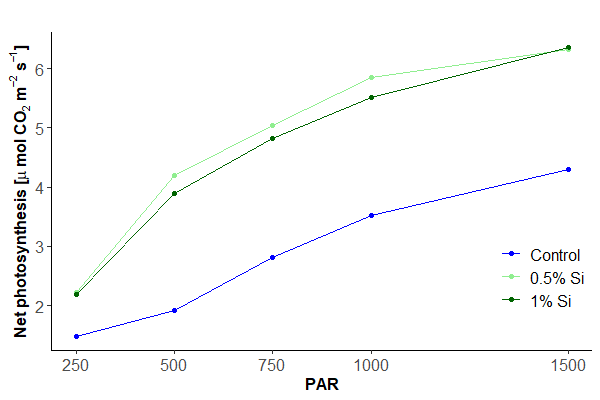


**FIGURE S3 Mean long term measurements of NEE for the different treatments (control, 0.5% ASi and 1% ASi) at different light intensity. Data from GFS-3000 measurements at the leaf level.**

**FIGURE S4 Soil CaCl_2_ and ASi concentration for the different treatments at tillering and grain filling stage.**
